# Development and Implantation of a Universal Talar Prosthesis

**DOI:** 10.3389/fsurg.2019.00063

**Published:** 2019-11-19

**Authors:** Julia Bowes, Samer Adeeb, Andrew Grosvenor, L. Beaupre, Nadr M. Jomha

**Affiliations:** ^1^Department of Surgery, University of Alberta, Edmonton, AB, Canada; ^2^Civil and Environmental Engineering, University of Alberta, Edmonton, AB, Canada; ^3^iRSM, Misericordia Hospital, Edmonton, AB, Canada; ^4^Department of Rehabilitation Medicine, University of Alberta, Edmonton, AB, Canada

**Keywords:** talar replacement, avascular necrosis, talar prosthesis, universal talar replacement, synthetic bone

## Abstract

Talar avascular necrosis (AVN) can result in bone collapse with subsequent ankle and subtalar osteoarthritis ending in significant pain and disability. Custom talar body prostheses have been implanted with good results but these are difficult to design, costly and require extensive planning. In the past few years, we have investigated the feasibility of a universal talar replacement prosthesis through multiple studies. This report documents that development and the results from the first patient to receive a universal talar replacement prosthesis. A patient with bilateral talar AVN with collapse had implantation of two universal talar prostheses with final evaluations at 34 months (right) and 12 months (left) post-implantation using visual analog scale, range of motion, SF-36 questionnaire, and personal reflection. The patient had decreased pain, increase range of motion, improvement (or no change) on all domains of the SF-36 and expressed great appreciation for having the procedures done. This report demonstrates the effectiveness and feasibility of a universal talar prosthesis. Continued development of this type of implant can decrease costs, improve access, and provide an acceptable alternative when a custom prosthesis is not possible.

## Introduction

The talus has relatively poor blood supply in comparison to other articular bones due to its lack of muscular and tendinous attachments. As a result, the talus is particularly susceptible to avascular necrosis (AVN). Talar AVN can be due to multiple causes including trauma, steroid use, metabolic, or idiopathic among other causes. Talus AVN with collapse leads to ankle and subtalar joint incongruity resulting in pain, stiffness, and disability. Due to the lack of effective treatment options, talar AVN with collapse is an extremely difficult problem. Once AVN with collapse occurs, total ankle arthroplasty becomes contraindicated (1) and, while bracing can provide some temporary relief, definitive treatment is typically tibio-talar-calcaneal fusion. This procedure can provide reasonable pain relief, but comes at the expense of loss of ankle and hindfoot motion resulting in difficulties ambulation with increased stress at surrounding joints (2, 3).

Since 1974, talar body prosthesis of various designs have been employed in select cases and all of these have been custom-made based on the contralateral talus (4–6). A group from Thailand custom milled 17 talar body prostheses out of stainless steel using slit scanograms (7). Their initial results were promising. They continued to implant more prostheses and the 10–36 year follow-up of a larger cohort showed surprisingly good results with 28 of 33 prosthesis still *in situ* despite many years of use (8). The next most comprehensive study was by a group in Japan that reported on 22 replacements in 2012 (9) followed up by 55 replacements in 51 patients in 2015 (10). These patients also reported good results with all prostheses remaining *in situ*. At 24–96 month follow-up they had significantly decreased pain and increased function scores with no signs of instability.

It appears that a custom-made talar prosthesis is a viable treatment option for select patients with talar AVN. To date, *all* of the reported results have used *custom-made* prostheses, which required the contralateral talus to template the design of the talus being replaced. We have also performed this multi-step procedure, which involves obtaining high definition CT scan with 1 mm cuts of the opposite talus, inverting the image, segmenting it, developing an. stl file and then either milling or 3D printing the talar prosthesis to develop the final product. It also requires expertise to create the .stl file and identifying a manufacturer who can produce the final product at medical grade for implantation. Clearly this is possible as noted by the published studies, but historically it has been complex, time consuming and traditional subtractive manufacturing techniques, such as multi-axis CNC milling, can be very expensive due to CNC programming and tooling, fixturing, surface finishing on low volume items. That said, more recently, additive manufacturing such as 3D printing has changed these parameters significantly. Another consideration is that the custom-made application is limited to single sided talar AVN because there is no clear template if the opposite talus is affected.

Interest in the development of a universal talar replacement was stimulated by the reality that the only real alternative surgical treatment for talar avascular necrosis with collapse would be a tibio-talar-calcaneal fusion that results in severe ankle and hindfoot stiffness and poor functional outcomes (11). To investigate this possibility, we first looked at 24 tali CT scans randomly selected and compared the overall shape. It was determined that the overall shape of the tali was very similar and the difference was primarily a matter of scale (12). As most of the CT scans were from male patients, we conducted an expanded study of 91 randomly selected CT scans (50 male and 41 female) and compared the shape of male and female tali. We determined that there were no specific sub-shapes of tali (i.e., all were similar shapes) and that male and female tali were a very similar shape (with a smaller average deviation between sexes than within sexes). It was concluded that a 10-implant range of sizes would fit the vast majority of patients with minimal deviation from their normal joint contours (13). Using this unexpected finding, we then completed a cadaveric study using the method of sizing that will be described in this paper. That paper documented acceptable size matching and joint congruity using when universal prostheses were implanted in cadaveric ankle joints (14). This series of work was performed in conjunction with the implantation of prostheses reported here in this case report. By comparing the 91 tali with each other, after scaling to the same volume, we identified the average shape by finding the one talus that has the least deviation when compared to the remaining tali (13). Then we applied some custom modifications to ease the insertion to develop the universal talus prosthesis. We have subsequently used this method with an excellent fit in a patient with a pre-existing fusion on one side and talar AVN with collapse on the other side.

This case report details the clinical outcome of implantation of two *universal* (total talar replacements) TTR in one patient for the treatment of bilateral talus AVN. A custom implant could not be modeled for this patient because both tali were collapsed. To our knowledge, this case is the first reported universal talar prosthesis and its associated clinical outcomes.

### Case Report

**Figure 1:**
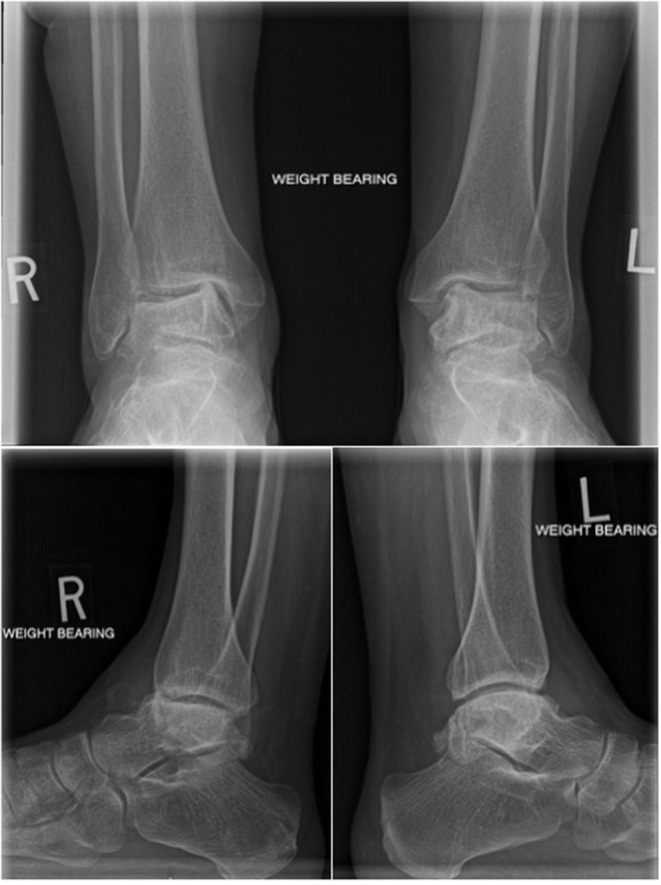
Radiographs at the time of presentation including bilateral AP weightbearing, right ankle weightbearing lateral and left ankle weightbearing lateral views.

The patient was a 54-year-old female with bilateral ankle pain and stiffness due to talus AVN secondary to corticosteroid use for Crohn's disease. The patient had exhausted all non-operative treatment modalities. The patient's right ankle was addressed first because it was more symptomatic. A tibio-talo-calcaneal fusion was considered; however, given her age, bilateral disease, and the desire to maintain ankle range of motion, a TTR was the treatment of choice. At presentation to the operating surgeon, the patient had collapse and deformity in both tali (Figure 1). As a result, using a custom implant modeled on contralateral talar CT measurements was not possible; therefore, a universal implant was created as described below.

Explicit informed written consent in accordance with the Declaration of Helsinki of the experimental nature of the procedure was obtained from the patient and ethics approval was obtained from the University of Alberta Ethics Review Board (Pro00016698). Health Canada approval was obtained for implant insertion. Outcomes including range of motion (ROM), pain scores, and SF-36 were obtained at the following intervals: pre-operative right TTR, 6 months post-operative right TTR, 22 months post-operative right TTR/pre-operative left TTR, 28 months post-operative right TTR/6 months post-operative left TTR, and 34 months post-operative right TTR/1 year post-operatively left TTR.

Pain was recorded using a visual analog scale (VAS) in which zero represented no pain and 10 the worst pain. The SF-36, a validated generic health status questionnaire, was also used. The SF-36 consists of eight health dimensions that can be compared to age and sex population means. A score of zero is equivalent to maximal disability and a score of 100 is equivalent to no disability. In addition, the patient was asked three questions to determiner her overall perceptions of her talar replacements in terms of impact to her foot and ankle specific function and overall quality of life.

### Universal Implant Generation

Three implants were created prior to the patient undergoing the first (right) TTR (Table 1).

**Table 1:** Summary of talar prosthesis design.

**Implant name**	**Design**
Custom	Inverted left talus with shaping for dome to fit plafond and fibula
Universal	Size 4 implant generated from previous research
Universal modified	Same as Universal but modified by customizing small sections in attempt to better align joint surfaces

#### Custom

The CT scans of the patient's bilateral tali, tibia, and fibula were used to create a “custom” implant of the talus by extracting the three-dimensional CAD files of the patient's bilateral tali bones from medical CT data using Mimics v 18 software (Materilise, Leuven, Belgium). The resultant 3-D digital data was imported into Freeform Modeling Plus software (3D Systems, Rock Hill, South Carolina, USA). The volume of the developed talus model was calculated. To account for the defect in the talar dome, the dome shape was further adjusted to customize the fit to articulate with the patient's tibial plafond and fibula articulating surfaces obtained from the CT scan. Once finally adjusted to articulate with the surrounding bony structures, an. stl file was generated. The .stl file was 3D printed by Southern Medical Pty in South Africa. This was considered the “custom” implant (but not truly custom because the important talar dome portion was not custom as described).

#### Univsersal

The universal implant was created from the file generated after a geometric research study completed earlier (13). That study determined a 10-size implant range to cover the possible options based on the volume of the talus. The volume of the “custom” implant was determined from the CT scan imaging (consisting of affected talus plus an estimate of the missing portion of the dome of that talus). This information was used to select a size 4 implant and this was considered the “universal” implant.

#### Universal Modified

Finally, the third implant was created by modifying the “universal” implant on Freeform Modeling Plus software performed by the operating surgeon to shape the implant surface relative to the surrounding bones to see if it would improve the fit. This was considered the “universal modified” implant.

**Figure 2:**
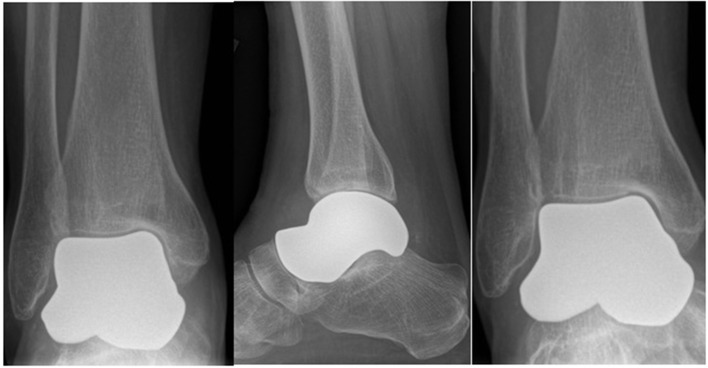
Post-operative 6 month radiographs (AP, lateral and mortise) of right total talus replacement.

The first (right) implants were printed in a titanium alloy (Ti 6Al 4V) with a titanium nitride coating applied. For the second (left) talus, the implants were generated using the .sstl files for the “universal” and the “universal modified” inverted and then printed in cobalt chrome. No further customization for the left was done because the right “universal” implant fit well and the patient was doing well clinically (Figure 2).

### Clinical Implantation

#### Surgical Procedure for Right Talus

The talus was exposed through an anterior approach and the talus was removed in a piecemeal fashion. The articular surface of the subtalar joint was well-preserved. On the tibial plafond side, there was full-thickness wearing of the cartilage on the anteromedial corner. The remaining cartilage was in reasonable condition, especially posteriorly. The medial and lateral malleolar cartilage was also in reasonable condition. The three implants were trialed with the primary surgeon blinded to which implant was being inserted. After insertion, each implant was examined under direct vision and using fluoroscopy. Clinical examination included fit within the ankle joint as well as range of motion of the ankle, subtalar, and talonavicular joints. Fluoroscopic examination included joint congruency and overall positioning with the remainder of the foot. All three implants fit well; however, the universal implant with no modification was selected to be the best fit clinically and fluoroscopically (Figure 2). After final implant selection and insertion, the anterior capsule and extensor retinaculum were repaired. Post-operatively the patient was placed in a plaster posterior slab and kept non-weight-bearing for 2 weeks. She was allowed to progressively weight-bear at 2 weeks in an aircast followed by full weight-bearing without immobilization at 6 weeks post-operatively. The patient was allowed range of motion exercises starting at 2 weeks post-operatively.

**Figure 3:**
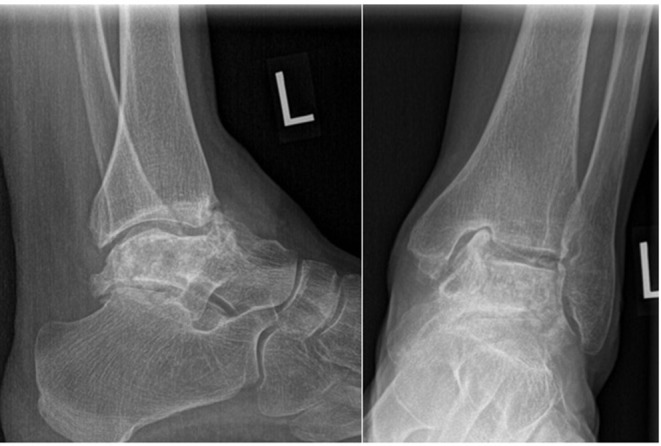
Pre-operative left ankle radiographs. Note the severe collapse since the previous radiograph and groove in the medial corner of the tibial plafond.

Twenty-two months, later the patient underwent a TTR on the left side. Unfortunately, given the delay for numerous reasons, the talar deformity had progressed resulting in a groove being worn into the medial side of the tibial plafond (Figure 3).

Given that the patient did well clinically, it was decided to use the same size matched universal implant but this one was cobalt chrome (see Discussion). This was performed using the same image processing file as for the right side but inverted for a left talus.

### Surgical Procedure for Left Talus

**Figure 4:**
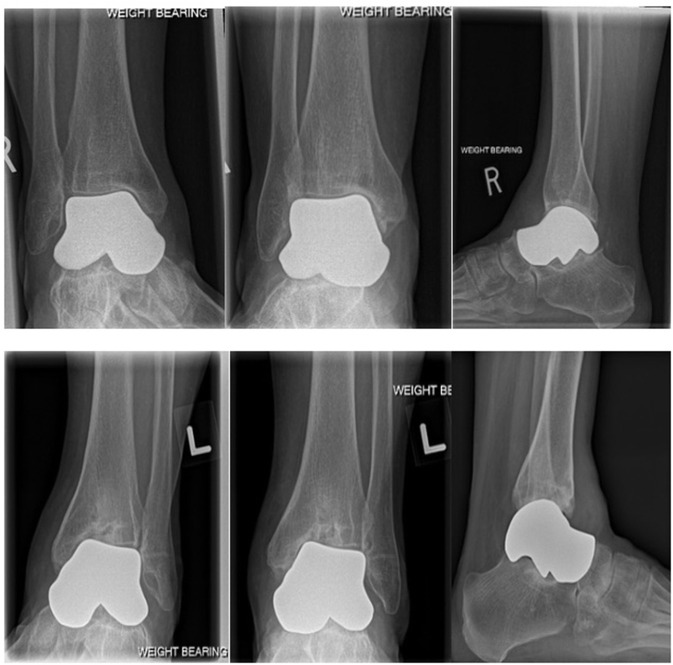
**Top row**: Weightbearing radiographs (mortise, AP and lateral) 34 months post-operative right total talus replacement. **Bottom row**: Weightbearing radiographs (mortise, AP and lateral) 1 year post-operative left total talus replacement. As noted in the preoperative radiograph, the medial corner of the tibial plafond was compromised resulting in tilting of the prosthesis in the ankle mortise.

The surgical approach for the left talus was the same as the right talus with the differences between the two procedures noted here. There was a groove in the medial corner of the tibial plafond so a small osteotomy was performed and the subchondral bone was punched down to improve the contour of the articular surface. Osteophytes were resected off the anterior tibia. A universal cobalt chrome total talar prosthesis was implanted. The fit of the prosthesis was confirmed clinically and radiographically (Figure 4, bottom row). A suture anchor was used to augment the anterior capsule repair. The same post-operative rehab protocol was followed.

The last patient clinical follow-up was at 34 months for the right talus and 12 months for the left talus (Figure 4).

## Results

For the right TTR after 34 months, ROM increased from 5→ 10 degrees of dorsiflexion and from 30→ 40 degrees of plantarflexion. After 28 months, pain decreased on the VAS in all three conditions (rest 4→ 2; activity 10→ 5, night 8→ 6). Improvement in pain scores were noted soon after the surgery but continued to improve with the best results noted at the 28 month post-operative visit. The 34 month pain results were not included as the patient stated she was having a severe flare of her Crohn's disease and could not differentiate her ankle pain from her whole body pain.

For the left TTR after 12 months, ROM increased from 0→ 10 degrees dorsiflexion and stayed at 45 degrees plantarflexion. After 6 months, pain had decreased in 2 of 3 conditions (rest 10→ 3; activity 10→ 10; night 10→ 6). Once again, the 12 months pain results were not included due to the Crohn's disease flare up she was experiencing at the time.

A summary of the SF-36 scores is seen in Table 2. The patient was asked specifically to take the effect of her TTR into account. There is a trend of improvement from pre-operative SF-36 data vs. the data after the patient had both tali replaced in most categories. Prior to any operative intervention on either ankle, the patient's physical functioning scored 15 vs. 30% at the most recent follow-up. During this same time period the patient's energy/fatigue went from 5 to 15%, social functioning from 37.5 to 62.5%, and pain from 10 to 45% with higher percentages indicating less dysfunction. Role limitation due to physical health showed no change throughout the entire time period with the patient reporting maximal disability in this category. Conversely, neither role limitation due to emotional problems and emotional well-being changed with the patient reporting no role limitation due to emotional problems and stable emotional well-being throughout the follow-up period. As can be noted, the best scores were at 28 months post-right TTR/6 months post-left TTR due to the flare of her Crohn's disease at the 34/12 month visit.

**Table 2:** SF-36 scores for each of the eight scaled scores.

	**Right pre-op (%)**	**Right 6 months post-op (%)**	**Right 22 months post-op, Left pre-op (%)**	**Right 25 months post-op, Left 3 months post-op (%)**	**Right 28 months post-op, Left 6 months post-op (%)**	**Right 34 months post-op, Left 1 year post-op (%)**
Physical Functioning	15	25	5	10	35	30
Role limitations due to physical health	0	0	0	0	0	0
Role limitations due to emotional problems	100	100	100	100	100	100
Energy/fatigue	5	25	15	20	45	15
Emotional well-being	84	92	80	84	80	88
Social functioning	37.5	75	12.5	62.5	75	62.5
Pain	10	0	22.5	45	45	45
General health	35	40	30	40	70	50
Health change	50	25	50	75	75	75

*A score of 0 is equivalent to maximum disability, a score of 100 is equivalent to no disability*.

In her semi-structured interview, the patient reported very positive perceptions of the surgeries on her ankle-specific function and quality of life. We generated an ankle specific self-reported satisfaction scale whereby, zero is the same as pre-operatively and ten is a “normal” functioning ankle. On this scale her right ankle scored an 8.5 and her left ankle scored a 5. See specific comments in Box 1.

Box 1 **Qualitative statements made by patient at most recent follow-up.**

**Table:** 

Are you better than prior to your surgeries?	Definitely. By far better.
How has the surgery affected your life?	I can walk again. So thrilled I had this opportunity to be able to walk normal.
Would you do it again?	Yes for sure.

No complications have occurred during the 34-month follow-up period from the right TTR and 1 year of follow-up from the left TTR. There were no intra-operative complications, neurovascular injuries, adverse events, readmission to the hospital, major bleeding, post-operative infections, or implant failure. At the most recent follow-up the radiographic appearance of the right implant is stable and seen in Figure 4 (top row).

## Discussion

To our knowledge this is the first report of clinical implantation of a universal talar prosthesis. Other case reports and series on talus replacements have utilized custom-made implants. The process of customizing an implant can be complex and costly. There are circumstances in which a normal talus from the patient is not available. The benefits of a universal talus replacement are that it would be immediately available (i.e., pre-made) and the direct implant cost would be low compared to a custom-made implant. Furthermore, trial implants can be created and used during the surgical procedure to identify the best sized implant. As this research is focused on the actual clinical applicability of a universal implant, it does not address the complexities and costs associated with developing an approved standard prothesis accepted by regulatory bodies such as the FDA.

This case study supports the use of a universal talar prosthesis in specific circumstances. This patient had improvement in multiple areas of her life as expressed on the SF-36. Unfortunately, circumstances around the prostheses insertion make objective measures difficult to accurately portray how the insertion of the implants have improved the patient's life because of the bilateral nature and ongoing poorly controlled systemic illness. Based on the primary surgeon's multiple interactions with the patient and her husband as well as her responses to questions about her function and satisfaction with the surgery, it appears that the VAS, ROM, and SF-36 scores (even though significantly improved) do not accurately reflect the benefit this patient has received from these surgeries. Furthermore, the bilateral nature of the disease with the delayed deterioration of the left side including the tibial plafond and the staggered implantation timing likely has led to the differences in scoring between sides. There is a reasonable chance that the left side will continue to improve as time passes.

This case study supports further investigation of a universal talus prosthesis, but some learnings were evident. The delay in performing the second side resulted in significant destruction of the medial corner of the tibial plafond due to further collapse of the talus with subsequent suboptimal positioning of the implant in the joint. This reinforces the importance of having readily available implants to prevent unnecessary delays and subsequent unnecessary joint destruction that can compromise long-term outcomes. The delay in this case was for two reasons. This was the first universal implant we had performed, and we wanted reasonable assurance that the patient would do well. There was some concern about titanium ion shedding with printed titanium therefore we changed to the harder cobalt chrome material. There were manufacturing delays due to the “custom” nature (meaning they were only printing one at a time) of the implant.

The clinical outcome of the participant presented in this case report demonstrates that a universal TTR provides good results with maintenance of range of motion and SF-36 scores better than historical results of tibio-talar-calcaneal fusion. Theoretically, a range of 10 implants sizes can be pre-made and be ready for immediate insertion at low cost with good functional outcomes. More investigation is required to confirm long-term outcomes and applicability across many cases in addition to understanding the economics of establishing this as a standard talar prosthetic implant.

## Data Availability Statement

All relevant datasets are presented in this study. For further details, please contact the corresponding author.

## Ethics Statement

The studies involving human participants were reviewed and approved by University of Alberta Ethics Review Board (Pro00016698). The patients/participants provided their written informed consent to participate in this study. Written informed consent was obtained from the individual(s) for the publication of any potentially identifiable images or data included in this article.

## Author Contributions

NJ and SA: generation of idea and prosthesis. JB and NJ: performance of surgical procedure. JB, LB, AG, and NJ: data analysis. JB, LB, AG, NJ, and SA: paper preparation and review.

### Conflict of Interest

Southern Medical Pty in South Africa helped with the production of these implants. The authors declare that the research was conducted in the absence of any commercial or financial relationships that could be construed as a potential conflict of interest.
